# Novel therapeutic strategies for osteoarthritis: from mechanistic insights to precision medicine

**DOI:** 10.1038/s41413-026-00540-6

**Published:** 2026-06-11

**Authors:** Muhammad Umar, Ziling Wang, Jingwen Li, Zhen Li, Gang Li, Liping Tong, Di Chen

**Affiliations:** 1 Faculty of Pharmaceutical Sciences, Shenzhen University of Advanced Technology, Shenzhen, China; 2 Center for AI-Driven Medical Research, Shenzhen Institutes of Advanced Technology, Chinese Academy of Sciences, Shenzhen, China; 3 AO Research Institute Davos, Davos, Switzerland; 4 Institute of Biomedicine and Biotechnology, Shenzhen Institutes of Advanced Technology, Chinese Academy of Sciences, Shenzhen, China

**Keywords:** Osteopetrosis, Metabolic bone disease

## Abstract

Osteoarthritis (OA) is a prevalent chronic degenerative joint disease that significantly impacts the quality of life for over 500 million individuals affected. OA is increasingly recognized as a whole-joint disorder characterized by complex biochemical and cellular changes across various joint tissues. However, traditional approaches such as nonsteroidal anti-inflammatory drugs (NSAIDs) and corticosteroids often fall short in halting disease progression or restoring joint function. Thereby, recent innovations in OA management are focused on underlying pathophysiology, driving the development of regenerative therapies, targeted anti-inflammatory agents and senolytic or senomorphic approaches. Accordingly, this paper reviews current progress in these areas by integrating evidence from clinical and pre-clinical studies to clarify therapeutic limitations, unresolved mechanistic and phenotypic gaps and the emerging strategies required to advance disease-modifying therapy in OA. It further outlines pathways toward precision, phenotype-aligned interventions, an integration that current literature has not yet consolidated.

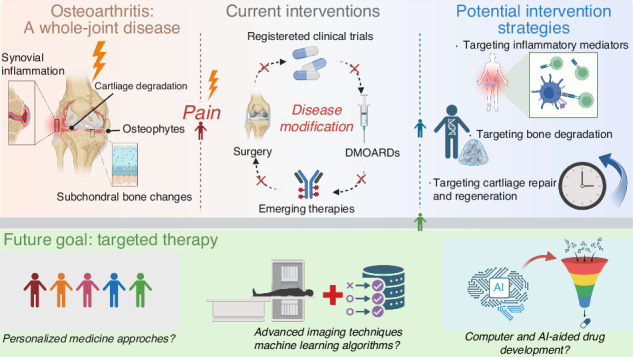

## Introduction

Osteoarthritis (OA) is the most common degenerative joint disease, affecting over 32.5 million individuals in the United States and an estimated 500 million adults worldwide. It is characterized by the progressive degradation of articular cartilage, subchondral bone, ligaments, and synovial tissues owing to mechanical overload, genetic predispositions, aging, obesity, and low-grade chronic inflammation.^1^ These factors disrupt the balance between catabolic and anabolic processes within joint tissues and lead to structural failure, together with functional impairment. Clinically, OA manifests as debilitating symptoms such as joint pain, stiffness, crepitus, and reduced mobility, significantly impacting the quality of life.^2^ The gradual onset of diseases often delays diagnosis and allows silent progression of joint damage before significant functional impairment becomes evident. Despite extensive research efforts aimed at elucidating pathophysiology, precise etiology of OA remains poorly understood and poses challenges for developing effective disease-modifying therapies.^3^ Since the prevalence of OA is increasing, particularly among aging populations, it leads to substantial economic burdens due to healthcare costs and lost productivity. As the population ages, the demand for effective treatments continues to grow, prompting extensive research into novel therapeutic strategies.^4^

Pathophysiology of OA is characterised by a multifaceted interplay of mechanical, biological, inflammatory factors. Potential risks that aid in the onset of OA include metabolic disorders, aging, joint injury, and genetic alterations. Mechanical stress on joints leads to cartilage wear and tear, triggering a cascade of cellular responses that include the release of pro-inflammatory cytokines and matrix-degrading enzymes.^5^ Genetic factors significantly influence the pathophysiology of OA, mainly through the roles of specific genes such as β-catenin, which is integral to Wnt signaling pathway. This pathway is crucial for maintaining cartilage homeostasis, and its dysregulation can lead to increased catabolic activity, resulting in cartilage degradation and contributing to OA progression.^6^ In contrast, FrzB, also known as sFRP3, is a Wnt signaling antagonist that binds to Wnt ligands, preventing their interaction with receptors and inhibiting β-catenin activation. In OA, dysregulated Wnt/β-catenin signaling contributes to cartilage degradation and abnormal chondrocyte hypertrophy, with elevated β-catenin promoting the expression of matrix-degrading enzymes like MMPs and ADAMTS. Reduced levels of FrzB are associated with OA progression, highlighting its protective role and potential as a therapeutic target.^7^ Moreover, in OA, disrupted TGF-β/Smad3 signaling contributes to disease progression. Reduced Smad3 activity is associated with increased chondrocyte hypertrophy, reduced production of cartilage matrix components (like collagen II and aggrecan) and elevated expression of matrix-degrading enzymes. Additionally, aberrant TGF-β signaling can promote osteophyte formation and subchondral bone sclerosis, further exacerbating OA.^8^ Restoring proper TGF-β/Smad3 signaling is considered a potential therapeutic strategy to prevent OA progression by preserving cartilage integrity and suppressing catabolic processes.^9^

Genome-wide association studies (GWAS) have identified numerous single nucleotide polymorphisms (SNPs) linked to OA risk and highlighted how genetic variations can predispose individuals to the disease by altering their responses to mechanical stress and inflammatory processes. For example, specific SNPs in *Axin2* have been shown to influence joint health by modulating the degradation of *Axin1* through tankyrase-mediated pathways which is critical for controlling β-catenin levels and Wnt signaling activity.^10^ Hormonal reproductive factors have also been linked to OA susceptibility, with studies indicating that variations in genes related to reproductive hormones may impact the risk of developing OA.^11^ Epigenetic modifications can interact with genetic predispositions throughout different life stages, potentially “unmasking” genetic risks during aging or following joint injury. For instance, the differential roles of *Axin1* and *Axin2* in the formation of β-catenin destruction complexes suggest that their stability is crucial for regulating Wnt signaling in context of joint health.^12^ This complex relation between genetic factors and environmental influences reveals the multifactorial nature of OA. Importantly, OA is increasingly recognized as a heterogeneous disease composed of distinct phenotypes. The inflammatory phenotype is defined by synovitis and elevated cytokines while the metabolic phenotype is associated with metabolic syndrome and systemic low-grade inflammation. The mechanical or biomechanical phenotype arises from joint injury, malalignment, or excessive load.^13^ An additional senescence-associated phenotype is characterized by the accumulation of senescent cells and their pro-inflammatory secretions. Identifying these phenotypes enables patient stratification and supports the development of targeted therapeutic strategies aligned with precision-medicine principles.^14^

However, traditional treatment strategies primarily focus on symptom relief through non-steroidal anti-inflammatory drugs (NSAIDs) and analgesics, thereby presenting the need for innovative treatment strategies to address underlying OA pathophysiology.^15^ As understanding of joint-level molecular pathways has expanded, current therapeutic development is targeting dysregulated signaling networks such as Wnt/β-catenin, TGF-β/Smad3, inflammatory cascades, and metabolic stress responses. Furthermore, novel pharmacological agents aimed at pain relief and inflammation control are also being evaluated in clinical settings. As researchers continue to explore new compounds and delivery methods, there is hope that future therapies will not only alleviate symptoms but also address the underlying biological mechanisms contributing to OA development.^16^ In this review paper, we will discuss the latest advancements in pharmacological therapies for OA treatment including both clinical and pre-clinical studies that target various molecules or signaling pathways that affect OA pathophysiology such as inflammation, cartilage degradation, and subchondral bone remodeling. By analyzing the mechanisms of action, efficacy, risk factors, therapeutic targets and safety profiles of these emerging treatments, we aim to provide a nuanced understanding of their potential roles in managing OA.

## Osteoarthritis pain: characteristics, structural changes and mechanisms

Pain is an early symptom of OA that often appears before any radiographic changes and tends to even worsen with the progression of a disorder. Generally, OA pain results from nociceptive mechanisms due to joint damage and inflammation, however, recent studies reveal a more complex interaction of factors.^17^ OA pain occurs via several interconnected processes including nociceptive pathways, peripheral sensitization, central sensitization, and bone-related mechanisms. In addition, psychosocial factors and genetic influences also affect pain sensitivity, perception, and progression of OA among individuals.^18^ The structures within the joint are richly supplied with nociceptors found in areas such as joint capsule, ligaments, menisci, periosteum, and subchondral bone.^19^ OA involves pathological changes throughout the joint including inflammation of synovium, degeneration of ligaments and menisci, remodeling of subchondral bone, and the progressive degradation of articular cartilage.^20^

### Structural changes in OA

Mechanical pain during movement is a key symptom of OA, however, the severity of pain often does not correlate with the degree of anatomical damage visible in imaging studies. Patients with OA frequently demonstrate lower pain thresholds to mechanical pressure indicating mechanical hyperalgesia at both the affected joints and remote areas compared to healthy individuals. This suggests that neuronal sensitization plays a crucial role in chronic pain experienced by OA patients.^21^ The pathological changes within joint lead to the release of pro-inflammatory substances such as prostaglandin (PG) E2 and nerve growth factor (NGF) which can sensitize peripheral nociceptive fibers and enhance pain signaling to the spinal cord.^22^ Increased nociceptive input further sensitize neurons in the dorsal horn contributing to the overall pain experience in OA. Additionally, there is evidence of increased nerve density in OA joints potentially due to nerve sprouting which may also influence pain perception.^23^ However, the precise impact of this increased nerve density on chronic OA pain remains to be fully defined. In OA, the degeneration of articular cartilage and the resultant inflammatory response trigger the release of pro-inflammatory mediators such as interleukin-1β (IL-1β), interleukin-6 (IL-6), tumor necrosis factor-alpha (TNF-α), and NGF.^24^ These substances promote inflammation as well as sensitize nociceptive pathways, leading to increased pain sensitivity, known as hyperalgesia, and pain from normally non-painful stimuli, termed allodynia. The nociceptive signals are transmitted primarily through Aδ fibers, which convey sharp, localized pain, and C fibers, which transmit dull, aching pain.^25^

**Fig. 1:**
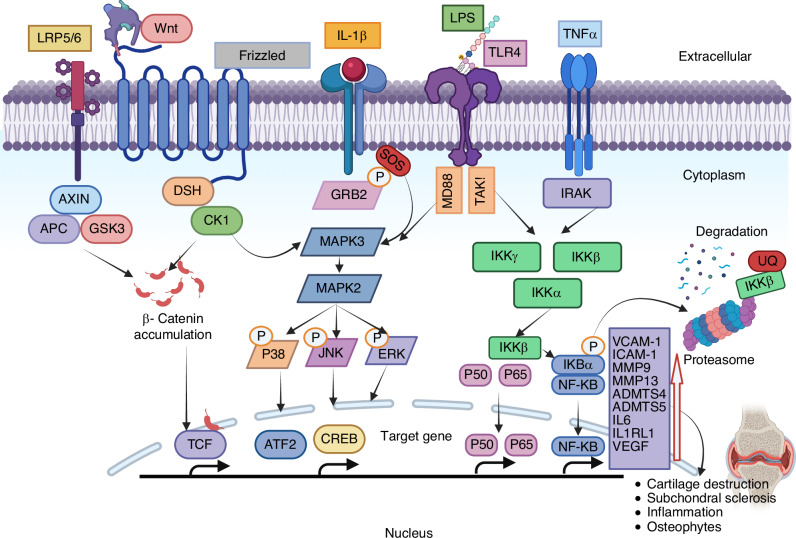
Key molecular pathways implicated in osteoarthritis involve NF-κB, MAPK, Wnt, and TLR signaling which collectively regulate inflammatory activation, catabolic enzyme expression, and cartilage matrix degradation. Abbreviations: NF-κB nuclear factor kappa-B, MAPK mitogen-activated protein kinase, Wnt Wingless-related integration site, TLR toll-like receptor, ERK extracellular signal-regulated kinase, JNK c-Jun N-terminal kinase, GSK-3β glycogen synthase kinase-3beta, DSH disheveled, IL-1β interleukin-1beta, MMPs matrix metalloproteinases

The activation of nociceptors increases firing rate of these sensory neurons that amplify pain signals sent to the spinal cord. As the condition progresses, continuous nociceptive input from the periphery can lead to central sensitization, which involves changes in spinal cord and brain, where repeated pain input enhances the transmission of pain signals in dorsal horn neurons. Neuroinflammatory substances, including TNF-α, BDNF, and various interleukins, are released from activated glial cells and contribute to increased responsiveness of neurons in central nervous system.^26^ The biomarkers involved in pathogenesis of OA are shown in Fig. 1 below. This heightened activity can result in long-term potentiation of synaptic transmission, making neurons more reactive to incoming pain signals. The interaction between peripheral and central sensitization not only amplifies pain signals but also alters the perception of pain, which ultimately makes it more chronic and challenging to manage. Additionally, hormonal factors such as cortisol and other stress-related hormones may further influence these sensitization processes and affect the overall pain experience in OA patients.^27^. Understanding these OA pain mechanisms has significant implications for treatment strategies. For instance, targeting inflammatory mediators can help reduce peripheral sensitization and alleviate pain. Similarly, therapies that focus on modulating central sensitization such as the use of certain analgesics or neuromodulators may enhance pain relief and improve patient outcomes. Thus, addressing both peripheral and central mechanisms is crucial for developing comprehensive treatment plans that can not only relieve pain but also improve overall joint function and quality of life for individuals with OA.

### Correlation between structural changes and pain

The exact correlation between structural changes and OA pain is complex and varies among individuals. Structural changes in OA such as cartilage loss, subchondral bone alterations, synovial inflammation, and osteophyte formation contribute to pain through mechanical stress, inflammation, and neural sensitization. However, the relationship is not straightforward because some individuals with significant structural changes report little to no pain, while others with minimal changes experience severe pain. Specific correlations include:

#### Subchondral bone

Bone marrow lesions (BMLs) and subchondral bone remodeling are strongly linked to OA pain, likely due to increased nerve density and inflammation in these regions. BMLs are localized areas of increased bone turnover and edema in the subchondral region that reflect active joint damage and strongly correlate with pain and structural progression in OA. Mechanically, this is driven by an acidic microenvironment and factors produced by bone cells. Then, bone-resorbing osteoclasts secrete factors like Netrin-1 and NGF, which directly activate acid-sensing ion channels and induce sensory nerve innervation in the subchondral bone, driving central pain signaling.^28^

#### Synovial inflammation

While structural changes on radiographs (joint space narrowing, osteophytes) show only a weak correlation with pain, MRI studies have consistently identified synovitis as a significant driver of pain.^23,29,30^ It works by releasing pro-inflammatory mediators that sensitize nociceptors as discussed in “Structural changes in OA”.

#### Cartilage loss

Although cartilage is denervated and cannot directly cause pain, its loss increases mechanical stress on subchondral bone and other innervated structures, indirectly contributing to pain as discussed in above section.

#### Osteophytes

Osteophytes can cause pain if they impinge on surrounding tissues or nerves, but they are not consistently associated with pain in all cases. Despite these associations, pain in OA is also influenced by non-structural factors such as central sensitization, psychological state, and systemic inflammation, which can amplify or modify the perception of pain. This multifactorial nature makes the correlation between structural changes and OA pain highly variable.^31^

### Osteoarthritis in specific joints

OA is a heterogeneous disease affecting multiple joints, including knee, hip, hand, and other joints.^32^ In this review, we focus on two typical forms of OA: knee osteoarthritis (KOA) and interphalangeal osteoarthritis (IPOA). KOA is the most prevalent form of weight-bearing joint OA, while IPOA is a paradigmatic example of a non-weight-bearing joint, exhibiting a robust genetic predisposition. This section facilitates the discernment of the impact of non-mechanical factors, including genetics, metabolism, and systemic inflammation. By comparing and contrasting these two forms, it is possible to gain deeper insights into the distinct roles of mechanical stress, aging, metabolism, and genetic influences in the pathogenesis and clinical management of OA.

#### Knee osteoarthritis (KOA)

KOA is a degenerative joint disease specifically affecting the knee joint, a major weight-bearing synovial joint. KOA has been among the top 20 causes of global disability over the past decades.^33^ The prevalence of KOA has increased by 2.1-fold since the 1950s, a trend largely attributed to the rise in both life expectancy and body weight.^34^ While etiology is multi-factorial, risk factors such as obesity, previous knee injury, aging, and genetic predisposition are well-established contributors to disease initiation and progression. Especially, obesity and knee injury are two major risk factors of KOA.^34^ These factors promote KOA development partly by inducing abnormal mechanical loading on knee joints, leading to articular cartilage wear and ligament damage, which ultimately culminates in the development of KOA. However, the occurrence of arthritis in non-weight-bearing hand joints among obese patients suggests that obesity influences joints through mechanisms beyond mere mechanical loading.^35^ Furthermore, the onset of KOA in elderly patients without obesity or knee injury underscores that KOA is a paradigm of the interplay between mechanical stress and systemic biological factors.^36^ A deeper understanding of these interactions is therefore crucial for developing therapies that can halt disease progression rather than merely alleviate symptoms. In contrast, IPOA, as a non-weight-bearing joint disease, provides a unique perspective for studying the role of intrinsic factors such as genetics and metabolism in OA.

#### Interphalangeal osteoarthritis (IPOA)

IPOA refers to the degenerative joint disease specifically affecting the interphalangeal joints of the fingers and toes, including both the proximal interphalangeal (PIP) and distal interphalangeal (DIP) joints. IPOA is a localized form of OA, characterized by the progressive breakdown of articular cartilage, subchondral bone remodeling, osteophyte formation, and varying degrees of synovial inflammation. Clinically, it presents with joint pain, stiffness, swelling, and reduced mobility, often leading to functional impairment and decreased quality of life. The condition is more prevalent among older adults and is especially common in postmenopausal women. Although the exact etiology remains multifactorial, risk factors such as age, genetic predisposition, and previous joint injury contribute to disease progression. Radiographically, IPOA is marked by joint space narrowing, subchondral sclerosis, and the presence of Heberden’s nodes at the DIP joints and Bouchard’s nodes at the PIP joints.^37^

In IPOA, metabolomic studies have revealed dysregulation in several key metabolic pathways, notably those involved in energy metabolism, amino acid metabolism, lipid metabolism, and responses to oxidative stress. One of the primary findings is the alteration in tricarboxylic acid (TCA) cycle intermediates, indicating mitochondrial dysfunction and impaired energy production in chondrocytes. This metabolic shift contributes to cartilage degradation due to reduced synthesis of extracellular matrix components and increased apoptosis of chondrocytes.^38^ Furthermore, disturbances in amino acid metabolism, particularly involving glutamine, arginine, and proline, suggest an imbalance in protein turnover and collagen remodeling, processes critical for maintaining cartilage integrity. Increased levels of branched-chain amino acids (BCAAs) have also been linked to pro-inflammatory states and may exacerbate joint inflammation.^39^ Lipid metabolism, especially involving phospholipids and sphingolipids, is another crucial aspect. Metabolomic profiling has shown altered concentrations of lysophosphatidylcholines (LysoPCs) and ceramides, which are associated with inflammatory processes and cartilage catabolism. These lipids can modulate the activity of cytokines and matrix-degrading enzymes such as MMPs, accelerating cartilage breakdown. Additionally, oxidative stress plays a significant role, as reflected by elevated markers of oxidized lipids and reactive oxygen species (ROS) in the synovial fluid and serum of IPOA patients. This oxidative environment promotes further cartilage damage and perpetuates inflammation.^40^

## Current clinical strategies and therapeutic trials of osteoarthritis

OA remains a significant challenge in clinical practice due to its complex nature and lack of effective disease-modifying therapies. Recent research has highlighted the landscape of registered clinical trials, revealing that over 255 studies are currently investigating various pharmacological interventions, including potential disease-modifying OA drugs (DMOADs). DMOADs are therapeutic agents designed to cease, or reverse the structural progression of OA by targeting underlying pathogenic mechanisms rather than providing only symptomatic relief.^31^ Evaluations of these trials indicate that while some candidates such as sprifermin and BMP-7 have shown promise in improving cartilage thickness or pain relief, many have failed to demonstrate clinically meaningful outcomes due to the heterogeneity of OA and the inadequacy of trial designs.^3,41^ The development of DMOADs is crucial as these agents aim to modify the underlying disease process; however, no DMOADs have received regulatory approval yet. Table 1 shows the detailed summary of current therapeutic targets to address diverse disease phenotypes in OA clinical trials. Despite these advancements, significant gaps remain in current research, particularly regarding identifying specific patient phenotypes that could benefit from tailored therapies and the need for robust clinical trial methodologies that adhere to established guidelines. Current management strategies primarily involve symptomatic treatments such as NSAIDs and corticosteroids; however, these approaches often come with side effects and limited long-term efficacy, highlighting the necessity for more targeted interventions that address the multifaceted nature of OA.^42,43^

**Table 1:** A detailed summary of current therapeutic targets to address diverse disease phenotypes in OA clinical trials

Therapeutic agents	Route of administration	Phase	Sample size	Results	Trial id
A. Treatments targeting bone degeneration					
Strontium ranelate	Oral	KOA	30	Results not yet published	NCT03937518
Strontium chloride Hexahydrate	Topical	III (KOA)	300	Results not yet published	NCT00954629
Denosumab	Subcutaneous	II (IPOA)	100	Joint remodeling and prevention of new erosive joints	NCT02771860
Vitamin D3	Oral	IV (KOA)	137	Higher KSS score (50.89) showed positive effects clinically	NCT04177758
Calcitonin	Oral	III (KOA)	1 176	Reduced JSW score	NCT00486434
B. Anti-inflammatory and immunomodulatory agents					
Ibuprofen (800 mg)	Oral	I (KOA)	33	Minimized pain perception in treated group than placebo group	NCT00565084
Celecoxib	Oral	II	125	Reduced WOMAC scores and pain perception	NCT01341405
Celecoxib	Oral	III (KOA)	380	Reduced inflammation	NCT02688400
Hydroxychloroquine/ Atorvastatin	Oral	II (KOA)	21	Participants still showed moderate levels of synovitis	NCT01645176
Infliximab	Intra-articular	IV (KOA)	16	Enhanced anti-inflammatory effects with significant pain relief	NCT01144143
Canakinumab	Intra-articular	IV (KOA)	169	Reduced pain scores	NCT01160822
Tanezumab	Intravenous	III (KOA)	849	Significant anti-inflammatory response and reduced pain	NCT00863304
Fasinumab	Subcutaneous	III (KOA)	3 307	Improved OA conditions and significant reduction in pain	NCT03161093
Naproxen	Oral	KOA	223	Reduced WOMAC scores and enhanced functionality	NCT00790985
C. Cartilage repair and regenerative therapies					
Hyaluronic acid	Intra-articular	-	38	Enhanced viscoelastic properties and mobility	NCT01920152
	Intra-articular	IV (Glenohumeral OA)	70	Improved mobility and reduced pain	NCT02984228
Platelet rich plasma	Intra-articular	-	543	Reduced pain and stiffness as well as improved functional capacity	NCT01782885
	Intra-articular	II (KOA)	32	Improved mobility and reduced pain	NCT02958267
Chondroitin sulfate	Oral	III (KOA)	194	Reduced cartilage loss while enhanced mobility	NCT01354145
Chondrosulf	Oral	III (HOA)	163	Reduced cartilage loss and enhanced pain alleviation	NCT00291499
Chondroitin sulfate + glucosamine Hydrochloride	Oral	IV (KOA)	606	Significant cartilage repair was recorded with lowered WOMAC score	NCT01425853
TissueGene-C	Intra-articular	II (KOA)	102	Significantly higher IKDC scores and functionality	NCT01221441
Losartan	Oral	I/II	1	Enhanced cartilage repair, functionality, and pain alleviation	NCT04212650
MSCs	Intra-articular	III (KOA)	475	Improved regenerative properties and reduced pain	NCT03818737
	Intra-articular	KOA	75	Reduced pain while significant increase in regenerative properties	NCT03379168
D. Senolytic and senomorphic agents					
Fisetin	Oral	I/II (KOA)	75	Enhanced senolytic mechanism	NCT04210986
Atorvastatin	Oral	IV	198	Enhanced senomorphic mechanism of action and improved functionality	NCT01837069
	Oral	II (KOA)	21	Improved overall joint health	NCT01645176

*KOA* knee osteoarthritis, *HOA* hand osteoarthritis, *IPOA* inflammatory peripheral osteoarthritis, *MSCs* mesenchymal stem cells, *IKDC* International Knee Documentation Committee, *WOMAC* Western Ontario and McMaster Universities Osteoarthritis Index, *JSW* joint space width, *KSS* Knee Society Score

### Anti-inflammatory and immunomodulatory agents

These agents refer to therapeutic substances that modulate immune response and reduce inflammation to manage OA. They inhibit pro-inflammatory cytokines such as TNF-α and interleukins and affect signaling pathways like NF-κB and Janus kinase/signal transducer and activator of transcription (JAK/STAT).^44^ Their mechanisms also involve the regulation of immune cell activity, including T cell differentiation and macrophage polarization, which collectively contribute to the restoration of homeostasis in joint environment.

#### NSAIDs

NSAIDs are widely utilized in the management of OA due to their efficacy in alleviating pain and inflammation, primarily through the inhibition of COX enzymes, which are crucial for the synthesis of PGs, compounds that mediate inflammation and pain. By blocking COX-1 and COX-2, NSAIDs reduce the production of these inflammatory mediators, decrease pain perception, and improve joint function in OA patients.^45^ Collectively, NSAIDs represent the first-line strategy for targeting the inflammatory phenotype of OA by directly suppressing cyclooxygenase-driven prostaglandin pathways, forming a mechanistic foundation for more advanced anti-inflammatory interventions. Researchers investigated the efficacy of cuminaldehyde in combination with indomethacin for treating OA using monoiodoacetate (MIA)-induced rat model, which effectively replicated the disease symptoms and joint degeneration via COX-1 and COX-2 inhibition, leading to decreased IL-6 and IFN-γ while promoting the production of IL-10. Additionally, the action of indomethacin on phospholipase A2 further limited substrate availability for COX enzymes to modulate inflammatory pathways.^46^ To improve therapeutic efficacy of indomethacin, another study optimized the formulation of poly (lactic-co-glycolic acid) (PLGA) microparticles for intra-articular administration of indomethacin by adjusting the pH of the aqueous phase to 3, significantly enhanced the encapsulation efficiency (90%). The in vitro release study showed prolonged release over several days, confirming effective drug encapsulation in the PLGA MPs and inhibition of COX enzymes.^47^ These formulation innovations demonstrate delivery-based modifications capable of extending NSAID benefits beyond symptomatic relief by sustaining local COX suppression in inflamed joint tissues. Similarly, the innovative MMP13-responsive micro-nano hydrogel microsphere system was designed to optimize the efficacy of celecoxib, a COX-2 inhibitor, using cationic liposomes loaded with drug, allowing for controlled drug release in response to elevated MMP13 levels. Upon release, celecoxib inhibited COX-2, reduced the production of PGs, MMPs, and cytokines, specifically in areas with high MMP13 activity. This MMP13-triggered system links NSAID action to catabolic enzyme activity, illustrating an evolving trend toward phenotype-responsive delivery approaches.^48^ When the effects of viscosupplement delivering celecoxib were investigated in combination with hyaluronic acid (HA) and glycerol:sorbitol (GS) in a rat model of post-traumatic OA, reduced degeneration was recorded as lower Mankin scores were observed, particularly in the medial side of the tibia. The localized delivery of celecoxib inhibited COX-2 enhanced bioavailability as well as contributed to the lubricant properties beneficial for intra-articular therapy.^49^ When combined with HA and a low transition temperature mixture (LTTM) of glycerol and sorbitol, celecoxib showed improved solubility and bioavailability while mimicking the rheological properties of healthy synovial fluid. The results indicated that the GHA + CEX gel exhibited favorable shear-thinning behavior and appropriate viscosity for injection, which are essential for effective joint lubrication and immediate anti-inflammatory effects in the OA joint environment.^50^ However, when celecoxib was optimized with albumin nanoparticles (NPs), its efficacy increased compared to formulations using HA and GS. A biocompatible and colloidally stable delivery system was developed using human serum albumin NPs loaded with celecoxib, which demonstrated a higher encapsulation efficiency and sustained release profile. The NPs, with an average diameter of 72 nm, effectively penetrated OA cartilage and were internalized by primary human articular chondrocytes. Upon release, celecoxib inhibited COX-2, reduced PGE2 during in vitro assays, where celecoxib-loaded NPs significantly decreased PGE2 levels in chondrocytes and lipopolysaccharide-stimulated THP-1 monocytes.^51^ Together, these nano-delivery studies indicate that NSAIDs can be mechanistically refined to achieve site-specific modulation of inflammatory cascades, aligning with precision approaches for inflammatory-dominant OA. According to researchers, NSAIDs demonstrated varying efficacy for KOA patients in comparison to Gutong Patch (GTP). The study found that while both GTP and NSAIDs effectively reduced pain and improved quality of life, the selective COX-2 inhibitor (SCI) and the combination of GTP with SCI yielded better visual analogus scale (VAS) pain score improvements than GTP alone. Notably, NSAIDs provided faster pain relief and joint motion recovery compared to GTP. However, the combination of GTP and NSAIDs was less effective than NSAIDs alone, potentially due to the heat released from GTP interfering with pain relief in patients experiencing heat pain. Mechanistically, GTP likely exerted its analgesic effects through the modulation of inflammatory pathways, reducing the release of pro-inflammatory mediators, while NSAIDs primarily inhibit COX-1 and COX-2, thereby decreasing the synthesis of PGs that mediate pain and inflammation.^52^ Overall, GTP is suggested as a safer alternative for patients with KOA, particularly those with cardiovascular and gastrointestinal comorbidities, due to its lower incidence of gastrointestinal adverse events compared to NSAIDs. It came out that NSAIDs remain effective symptom-modifying agents, but their role within the broader mechanistic framework centers largely on inflammatory pathway suppression rather than comprehensive disease modification.

#### IL-1 Inhibitors

**Fig. 2:**
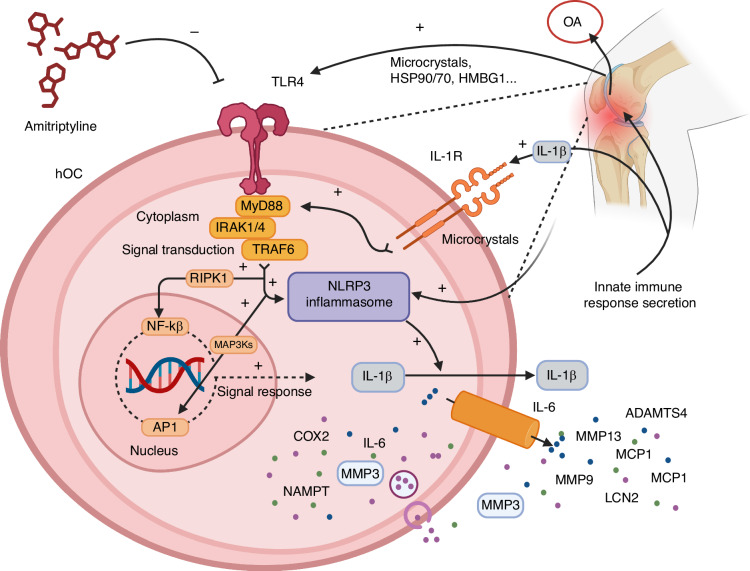
Amitriptyline modulates TLR4- and IL-1 receptor-mediated signaling in human osteoarthritic chondrocytes by influencing MyD88-dependent NF-κB and AP-1 activation, MAPK signaling, and the NLRP3/IL-1β inflammatory axis, collectively altering downstream cytokine and MMP responses. Abbreviations: MyD88 myeloid differentiation primary response 88, IRAK1/4 interleukin-1 receptor–associated kinase 1/4, TRAF6 TNF receptor–associated factor 6, RIPK1 receptor-interacting serine/threonine-protein kinase 1, AP-1 activator protein-1, MAP3Ks mitogen-activated protein kinase kinase kinases, ERK extracellular signal-regulated kinase, JNK c-Jun N-terminal kinase, NLRP3 NOD-, LRR- and pyrin domain–containing protein 3, COX-2 cyclooxygenase-2, NAMPT nicotinamide phosphoribosyltransferase, hOCs human osteoarthritic chondrocytes

**Fig. 3:**
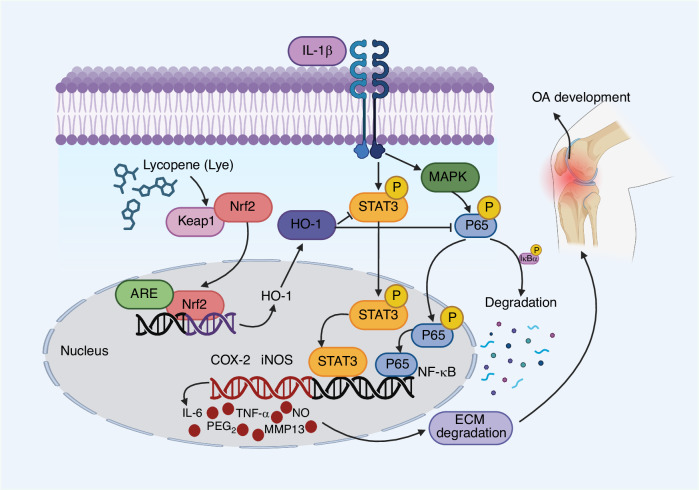
Lycopene regulates IL-1β–induced inflammatory signaling by modulating MAPK, STAT3, NF-κB, and Nrf2/HO-1 pathways in chondrocytes. Abbreviations: Keap1 Kelch-like ECH-associated protein 1, Nrf2 nuclear factor erythroid 2–related factor 2, HO-1 heme oxygenase-1, ARE antioxidant response element, STAT3 signal transducer and activator of transcription 3, P65 IκBα, inhibitor of kappa-B alpha, iNOS inducible nitric oxide synthase, NO nitric oxide, PGE₂ prostaglandin E₂, PLG₂ proteoglycan-2, ECM extracellular matrix

IL-1 inhibitors target IL-1R on synovial fibroblasts and chondrocytes to inhibit the binding of IL-1α and IL-1β. This blockade disrupts the activation of downstream signaling pathways, including the NF-κB and MAPK pathways, which are responsible for the upregulation of TNF-α, IL-6, and IL-8, as well as MMPs.^53^ Reduced expression of these inflammatory mediators and enzymes leads to a significant decrease in synovial inflammation and cartilage breakdown in OA conditions. These mechanisms position IL-1 inhibitors as central regulators of the inflammatory cascade that drives cartilage catabolism. He et al.^54^ investigated the efficacy and underlying mechanisms of Fengshi-Gutong capsule (FSGTC) as an IL-1 inhibitor in OA treatment and established an in vitro OA cell model using IL-1β to simulate inflammatory conditions. They revealed that FSGTC alleviated OA symptoms by upregulating long non-coding RNA PACER, which is involved in the regulation of COX-2 expression, reducing PGE2 levels and TNF-α and IL-1β, while promoting IL-4 and IL-10. Overall, IL-1-directed therapies suppress catabolic signals while shifting the joint microenvironment toward an anti-inflammatory state. Franco-Trepat et al.^55^ employed amitriptyline as an IL-1 inhibitor to discover its anti-inflammatory effects on innate immune responses in OA. Through a combination of in silico docking analysis, RT-PCR, siRNA, and clinical data mining, the study demonstrated that amitriptyline binds to TLR4, effectively inhibited TLR4- and IL-1 receptor-mediated signaling pathways in human OA chondrocytes, downregulated NLRP3 inflammasome expression, and diminished the production of IL-1β and stimulated anabolic effects within chondrocytes. This further supports the concept that targeting IL-1 signaling can simultaneously downregulate innate immune activation and promote cartilage-repairing pathways. The process and pathway are shown in Fig. 2 below. Su et al.^56^ used betulin to investigate its anti-inflammatory effects on cytokine inhibition, specifically IL-1β and TNF-α, in OA synovial fibroblasts and found effective in inhibiting mitogen-activated protein kinase (MEK), extracellular signal-regulated kinase (ERK), and NF-κB pathways due to the decreased phosphorylation of key signaling proteins. Betulin thus reinforces the broader mechanistic theme that IL-1 inhibition converges on suppressing MAPK and NF-κB-driven catabolism. Similarly, Zhan et al.^57^ investigated the anti-inflammatory effects of lycopene (Fig. 3) on OA using in vitro chondrocyte models and an in vivo mouse OA model, and found that lycopene increased the expression of Nrf2 and its downstream target HO-1, leading to inhibition of p65 nuclear translocation and reduced phosphorylation of STAT3. This results in the downregulation of COX-2, iNOS, PGE2, NO, TNF-α, and IL-6, as well as MMPs, ultimately mitigating extracellular matrix degradation (ECM). In the mouse OA model, oral administration of lycopene attenuated cartilage destruction and joint space narrowing, as evidenced by lower OARSI scores. In short, diverse IL-1 targeting agents suppress inflammatory transcriptional programs to preserve cartilage homeostasis.

#### IL-6 inhibitors

IL-6 inhibitors bind to IL-6R and prevent IL-6 from activating its receptor complex, which includes the signal transducer GP130. This blockade inhibits the activation of downstream signaling pathways, particularly JAK and STAT3 pathways, and reduces the expression of various pro-inflammatory cytokines and MMPs in OA patients.^44^ This therapeutic class aligns with strategies aimed at suppressing cytokine-driven inflammatory amplification that accelerates structural joint damage. In a retrospective analysis focusing on knee joint symptom alleviation, Murata et al.^58^ targeted synthetic disease-modifying drugs (DMARDs), particularly IL-6 inhibitors, such as tocilizumab and sarilumab, which inhibited joint destruction compared to other treatments like TNF inhibitors and cytotoxic T lymphocyte-associated antigen-4-Ig (CTLA4-Ig). DMARDs are therapies designed to halt the underlying disease process by targeting specific inflammatory or immune pathways. The underlying mechanism was the modulation of inflammatory pathways where IL-6 plays a critical role in promoting inflammation and joint damage. IL-6 promotes the activation of various signaling pathways, particularly through its receptor GP130, leading to transcription of pro-inflammatory genes and the release of additional cytokines that exacerbate OA symptoms. By inhibiting IL-6 signaling, these drugs reduced inflammatory response, lowered serum levels of C-reactive protein (CRP), and potentially mitigated pain associated with synovitis. Mima, et al.^59^ demonstrated that IL-6 activates the JAK2/STAT3 signaling pathway to increase production of inflammatory cytokines and hypertrophic markers *Col10a1* and *Runx2* in chondrocytes and *Runx2* contributes to cartilage degradation through upregulation of MMPs and ADAMTS. Thereby, they used Tyrphostin AG490 to effectively inhibit JAK2, blocks the downstream activation of STAT3, and alleviates spinal hyperexcitability induced by IL-6 ultimately improving ambulation and reducing pain mediators like bradykinin in experimental OA models. This mechanistic targeting positions IL-6 pathway inhibition as part of the broader JAK/STAT-modulating therapies that aim to limit catabolic signaling in chondrocytes. In this regard, dexamethasone is a significant therapeutic agent, while not directly inhibiting IL-6 and modulates IL-6 signaling pathways to effectively slow OA progression. Teng, et al.^60^ explored the effects of dexamethasone liposome (Dex-Lips) on alleviating OA induced by DMM in miR-204/-211-deficient mice. Dex-Lips inhibited of pro-inflammatory cytokines including IL-6, TNF-α, and IL-1β by promoting the polarization of macrophages towards the M2 phenotype while modulation occurred through the JAK2/STAT3 signaling pathway. Additionally, treatment with Dex-Lips enhanced glucocorticoid receptor (GR) expression in the knee joint, further contributing to its efficient anti-inflammatory effects. While, Chen, et al.^61^ evaluated the analgesic efficacy of a thermoresponsive polymeric dexamethasone prodrug (ProGel-Dex) in a mouse model of post-traumatic OA which showed anti-inflammatory and immunomodulatory effects via sustained release of low levels of dexamethasone which reduced expression of IL-6, TNF-α, and IL-1β through the inhibition of NF-κB and AP-1. Furthermore, ProGel-Dex promoted macrophage polarization towards the M2 phenotype in joints for 15 weeks of post-injection. These corticosteroid-based platforms extend the anti-inflammatory category by intersecting IL-6 modulation with macrophage reprogramming, emphasizing their role within the broader framework of immunoregulatory interventions.

#### DMARDs

DMARDs are a class of medications primarily used to treat inflammatory arthritis, including rheumatoid arthritis (RA), but their role in OA management is less well-defined yet. These drugs target TNF-α, IL-1, and IL-6, influence the activity of enzymes involved in inflammatory process including COX and lipoxygenase (LOX), slow down disease progression, and improve joint function.^62^ As therapeutic agents that directly modulate upstream inflammatory signaling, DMARDs align with OA mechanisms driven by synovitis, cytokine imbalance and immune-mediated cartilage degradation. However, the efficacy of DMARDs in OA management remains a subject of ongoing research. While they are not the first-line treatment for OA, some studies suggest that DMARDs may provide symptomatic relief and potentially slow the progression of joint damage in specific patient populations, particularly those with inflammatory features or coexisting conditions.^63^ In a multi-center, double-blind, randomized, placebo-controlled trial (RCT), Wang, et al.^64^ evaluated the efficacy of methotrexate in patients with hand OA (HOA) and synovitis. Methotrexate reduced pain and stiffness at 6 months via folate pathway inhibition, particularly dihydrofolate reductase, which decreases availability of purines and pyrimidines necessary for lymphocyte proliferation. This led to reduced synthesis of TNF-α and IL-1, and enhanced adenosine release, which has anti-inflammatory effects by activating adenosine receptors that inhibit further cytokine production. When evaluated in patients with KOA, a multi-center trial containing a total of 207 participants with symptomatic KOA were randomly assessed after receiving either oral methotrexate (10–25 mg weekly) or a matched placebo over 12 months while continuing their usual analgesia. Results showed that at 6 months, the methotrexate group experienced a significant reduction in average knee pain from a baseline of 6.4 to 5.1 on a numerical rating scale (NRS), compared to a decrease from 6.8 to 6.2 in the placebo group. Additionally, improvements were noted in knee stiffness and knee function, with significant differences favoring methotrexate in the Western Ontario and McMaster Universities Osteoarthritis Index (WOMAC) scores for stiffness (0.60 points) and function (5.01 points).^65^ These findings reinforce that methotrexate primarily intersects with OA pathophysiology by suppressing inflammatory mediators that contribute to synovitis-driven pain and cartilage catabolism. Several studies have been previously shown mechanisms of action of hydroxycholorquine (HCQ) in alleviating OA conditions and delaying progression via inhibition of TLRs and modulation of the immune response, reduction in TNF-α and IL-1, and downregulation of NF-κB, a key transcription factor involved in inflammation and cartilage degradation.^66^ However, OA-TREAT, an investigator-initiated, multi-center, randomized, double-blind, placebo-controlled trial evaluated the efficacy and safety of HCQ as a DMARD in patients with inflammatory and erosive HOA. However, there was no significant differences in pain or disability outcomes between the HCQ and placebo groups over 52 weeks, indicating that HCQ did not effectively modify the disease progression.^67^ It came out that the mechanistic influence of HCQ on TLR and NF-κB signaling may not sufficiently modulate the complex inflammatory phenotype of erosive hand OA. Zhao et al.^68^ utilized CCK-8 assays and Western blotting to evaluate the effects of sulfasalazine on FLSs, alongside a collagen-induced arthritis mouse model to assess its impact on joint inflammation and damage. The findings revealed that sulfasalazine treatment led to a decrease in ferroptosis-related proteins glutathione peroxidase 4 (GPX4), ferritin heavy chain 1 (FTH1), and solute carrier family 7 member 11 (SLC7A11). This indicated enhanced ferroptosis in fibroblast-like synoviocytes (FLSs) by downregulating PI3K-AKT-ERK1/2 signaling pathway and the activation of the P53 pathway which collectively contributed to reduced cell proliferation, migration, and invasion of FLSs. Additionally, sulfasalazine administration in the animal model alleviated synovial inflammation and joint swelling, further supporting its therapeutic potential. By targeting ferroptosis and PI3K-AKT-ERK signaling, sulfasalazine extends the DMARD category beyond classical cytokine suppression, relevant to inflammatory and metabolic stress-associated OA phenotypes. Overall, DMARDs are significant agents within OA therapy which play a role in modulating inflammatory pathways, immune activation and stress-response signaling.

#### TNF-α inhibitors

TNF-α inhibitors are a class of biologic agents that function primarily through the inhibition of TNF-α, a pro-inflammatory cytokine central to pathophysiology of OA and other inflammatory conditions. By blocking TNF-α, these inhibitors reduce the activation of NF-κB and decrease the production of IL-1 and IL-6.^69^ Furthermore, TNF-α inhibitors can also influence the activity of MMPs and enzymes that degrade cartilage to promote cartilage preservation and reduce pain associated with OA. As modulators of upstream inflammatory signaling, TNF-α inhibitors align with therapeutic strategies aim to interrupt cytokine-driven catabolism and protect joint tissues from chronic immune activation. Poutoglidou, et al.^70^ investigated the effect of chronic treatment with infliximab, a TNF-α inhibitor, on bone mineral density (BMD) and tendon inflammation in a rat model of collagen-induced arthritis. Infliximab treatment prevented arthritis-related conditions by directly neutralizing TNF-α and reducing inflammation. TNF-α inhibition also suppressed tendon inflammation by reducing levels of TNF-α and IL-23, improved trabecular microarchitecture and reversed pathological changes in tendons by modulating key pathways involving RANKL, Dkk1, sclerostin, and IL-23. When delivered via a thermosensitive hydrogel into the intra-articular space in vivo by Chen et al.^71^, infliximab led to downregulation of IL-1β, IL-6, and IL-17, which are involved in synovial inflammation, cartilage destruction, and bone degeneration. Specifically, IFX prevented the proliferation of synovial cells, inhibited leukocyte migration, and suppressed the production of tissue-degrading enzymes by neutralizing TNF-α. However, when administered in rat model induced by ACLT by Wen et al.^72^, IFX educed nociception and synovitis linked to the modulation of histone deacetylases (HDACs) and key regulatory factors in chondrocytes. IFX inhibited the expression of HDAC6 and HDAC7 which are associated with inflammation and cartilage degradation, while enhancing HDAC4 expression, which negatively regulates *Runx2*, decreased the activation of NF-κB, Jun N-terminal kinase (JNK), and ERK. In short, these findings demonstrated TNF-α inhibitors as agents that suppress inflammatory amplification loops, thereby integrating into the broader framework of treatments designed to curb cytokine-mediated joint destruction.

## Potential clinical interventions in osteoarthritis

OA continues to be a significant global health concern since recent studies indicate alarming trends in its prevalence and associated disability. According to the Global Burden of Disease Study 2019, OA significantly contributes to global morbidity. An estimated 528 million people worldwide are affected which reflects a 25% increase in prevalence from 1990 to 2019. OA ranks as the 11th leading cause of years lived with disability (YLDs) and accounts for approximately 14% of total YLDs due to musculoskeletal disorders.^73^ This rising prevalence of OA requires the development of effective treatments that go beyond symptomatic relief. Current pharmacological options such as NSAIDs and corticosteroids often fail to address the complex pathophysiology of OA which involves multifactorial mechanisms including inflammation, cartilage degradation, and subchondral bone remodeling.^74^ In this regard, clinical trials serve as a platform for evaluating innovative therapeutic agents such as DMOADs and biologics that target specific pathways implicated in OA progression. These targets represent the rationale for interventions aiming to modulate aberrant bone remodeling, suppress catabolic cytokine signaling, restore anabolic growth-factor balance or selectively eliminate senescent cells, thereby shifting treatment goals toward true disease modification. Recent advancements (Table 2) in trial methodologies including the incorporation of biomarkers and patient stratification based on OA phenotypes enhance the precision of treatment approaches and facilitate the identification of patient subgroups that benefit most from targeted therapies, thereby optimizing clinical outcomes.

**Table 2:** Recent innovations in OA treatment to enhance efficacy and therapeutic outcomes

Current treatments	Active agents	Route of administration	Type of study	Target joint	Efficacy	References
Biomaterials	Niacinamide and type II collagen	Oral	In vivo	Knee	Effective in alleviating joint pain and inflammation	^148^
	Hydrolyzed collagen	Intra-articular	Retrospective clinical trial	Knee	Effective strategy for relieving symptomatic OA conditions	^149^
	Chitosan	Intra-articular	In vivo	Knee	Protected cartilage destruction and relieved pain in OA rats	^150^
	Chitosan + Hyaluronic acid	-	In vitro	Knee, Hand	Reduced inflammation and oxidative stress with a positive impact on chondrocytes	^151^
	Gelatin + Eicosapentanoic acid	Intra-articular	In vivo	Knee	Delayed OA progression and OA-related inflammation	^152^
	PVA–PCL IPN Scaffolds	Surgical implantation	Pre-clinical	Knee	Effective against osteochondral defects	^153^
Nanoparticles and nanofibers	PLGA NPs + Hyaluronic acid	Intra-articular	Pre-clinical	Knee	Improved drug delivery and specificity to receptors for OA treatment	^154^
	PLGA NPs + Rapamycin	Intra-articular	Pre-clinical	Knee	Mitigated joint cartilage destruction, osteophyte formation, chondrocytes hypertrophy, synovial inflammation, and pain	^155^
	Hyaluronic acid-coated gelatin NPS + Kaempferol	Intra-articular	Pre-clinical	Knee	Reduced subchondral sclerosis, inflammation, and matrix degradation, while restoring cartilage thickness	^156^
	PLGA resveratrol NPs	Intra-articular	Pre-clinical	Knee	Inhibited apoptosis and promoted autophagy in OA conditions	^157^
	PLGA NPs + gold and methotrexate + anti-CD64 antibody	Intra-articular	Pre-clinical	Knee	Improved clinical indices and arthritic scores	^158^
	Tofa-*n*HA-GLT NPs	Intra-articular	Pre-clinical	Knee	Reduced inflammation and improved joint functionality	^159^
Stem cell and regenerative therapies	MSC/chondrocyte-based extracellular vesicles	Intra-articular	Pre-clinical	Knee	Delayed OA progression and promoted joint health	^160^
	Autophagic LC3^+^ calcified extracellular vesicles	-	In vitro	Knee	Reversed pathological calcification and degradation of cartilage	^161^
	Modified MSCs-derived extracellular vesicles	Intra-articular	Pre-clinical	Knee	Enhanced therapeutic efficacy and reduced injection frequency with improved bioavailability	^162^
	BMSCs-derived exosomes	Intra-articular	Pre-clinical	Knee	Promoted cartilage repair and ECM synthesis, as well as alleviated knee pain	^163^
Stimulus responsive drugs	ROS-responsive modified mesoporous silica NPs containing oltipraz	-	In vitro	Knee, Hand	Confirmed the great cartilage-protecting properties and reduced oxidative stress as well as toxicity	^164^
	Hypoxia and MMPs-Responsive Hydrogel containing hydroxychloroquine	Intra-articular	In vivo	Knee, Hand	Significantly attenuated oxidative stress and prevented cartilage from being destroyed	^165^
	pH-responsive MOFs system + hyaluronic acid loaded with protocatechuic acid	Intra-articular	Pre-clinical	Knee	Reduced synovial inflammation and improved joint functionality	^166^
	Thermo-responsive hyaluronan-based hydrogels + sulfo-dibenzocyclooctyne-PEG4-amine	Intra-articular	In vitro	Knee	Delayed OA progression and alleviated pain	^167^
Tissue engineering And 3D bioprinting	3D bioprinted micronized adipose tissue graft	Surgical implantation	Clinical trial	Knee	Lowered rejection response and better regenerative properties	^168^
	BMSC-laden 3D-bioprinted scaffold with MeHA/PCL + kartogenin and β-TCP	Direct implantation	Pre-clinical trial	Femoral trochlea	Improved joint function of the injured leg, reduced inflammation, and promoted cartilage defect repair	^169^

*BMLs* Bone marrow lesions, BMSCs Bone marrow-derived mesenchymal stem cells, NPs - Nanoparticles, *PLGA* Poly(lactic-co-glycolic acid), *HA* Hyaluronic acid, *PVA–PCL IPN* Polyvinyl alcohol-polycaprolactone interpenetrating network, *MeHA* Methacrylated hyaluronic acid, *β-TCP* β-tricalcium phosphate, *MOFs* Metal-organic frameworks, *DMOADs* Disease-modifying osteoarthritis drugs

### Treatments targeting bone degeneration

OA is characterized by complex changes in bone metabolism, structure, and function triggered by a combination of physiological factors such as age-related wear and tear, and genetic predisposition that influence joint health.^75^ A large number of OA-related studies focus on targeting subchondral bone degeneration; however, the involvement of bone structures extends beyond the subchondral regions. Different types of bones, such as subchondral bones, epiphyseal bone, periarticular bone, and carpal and metacarpal bones, are involved in hip, knee, periarticular, and hand OA, somehow affecting the bone-cartilage integrity and their ability to absorb physiological and non-physiological shocks.^76^ In early OA, accelerated bone remodeling leads to significant changes across various bone types such as subchondral bone thinning and increased porosity, while epiphyseal bones exhibit alterations in their growth plate architecture. Changes in periarticular bone density and structure affect joint mechanics due to localized bone loss and increased stiffness. Additionally, carpal and metacarpal bones showed increased trabecular spacing and reduced bone volume fraction.^77^ As OA progresses, all these bone types undergo compensatory remodeling; subchondral bone thickens and becomes sclerotic, epiphyseal bone may show signs of osteophyte formation, periarticular bone experiences increased mineralization, and carpal and metacarpal bones may develop cystic changes, all of which trigger cartilage degeneration and joint dysfunction.^3^ Several key treatments and drugs targeting OA-related bone mechanisms and metabolism have been tested via clinical trials, including anabolic agents, anti-resorptive agents, bone morphogenetic proteins (BMPs), osteogenic growth factors, vitamin D, and calcium supplements.

#### Osteogenic growth factors

Osteogenic growth factors are signaling molecules that stimulate the differentiation of mesenchymal stem cells (MSCs) into osteoblasts. MSCs are multipotent stromal cells capable of differentiating into cartilage, bone, and other connective tissues, making them a key regenerative cell type for repairing joint damage and restoring tissue homeostasis. These factors promote various biological processes, including cell proliferation, migration, and the synthesis of ECM components, which are essential for bone tissue development and repair.^78^ Common types of osteogenic growth factors, including BMPs, vascular endothelial growth factor (VEGF), and transforming growth factor β (TGF-β), have been shown to stimulate bone and cartilage anabolism in OA patients.

BMPs are a subgroup of TGF-β superfamily, primarily recognized for their roles in bone and cartilage formation. BMPs exert their biological effects through BMP receptor type IA (BMPR-IA), type IB (BMPR-IB), and type II (BMPR-II) which are serine/threonine kinase receptors located on the surface of target cells such as osteoblasts, chondrocytes, and MSCs.^79^ Upon BMP binding, these receptors activate the Smad proteins where phosphorylated Smad1, Smad5, and Smad8 translocate to the nucleus to regulate the transcription of osteogenic genes including *Runx2* and *Osterix*, promoting osteoblast differentiation.^80^ BMPs inhibit adipogenesis by downregulating peroxisome proliferator-activated receptor gamma (PPAR-γ) and its target genes.^81^ Additionally, BMPs enhance bone formation by promoting the expression of Wnt target genes and inhibiting the action of sclerostin, a Wnt antagonist.^82^ The targeting sites of BMP action include periosteum and endosteum where they stimulate osteoblast proliferation and differentiation while also modulating the activity of osteoclasts through factors like receptor activator of RANKL.^83^ BMPs regulate angiogenesis via endothelial cells with genes such as *Id1* being essential mediators of BMP-induced endothelial migration and tube formation.^84^ Li et al.^85^ explained that statins, particularly simvastatin, inhibited the synthesis of mevalonate to reduce the production MMP3 and MMP13, downregulated leptin expression in articular cartilage. This also triggered BMPs and inhibited osteoclastogenesis by blocking the RANKL-induced activation of NF-κB pathway, thus retarding subchondral bone deterioration induced by high-fat diet in mice. Bei et al.^86^ showed that BMPs inhibited cartilage lesions and micro-architecture deterioration of subchondral bone in patellofemoral osteoarthritic in ovariectomized rats with patella-baja by inhibiting osteoclast-mediated bone resorption and inhibiting MMP13 expression. Subsequently, a significant delay in the destruction of articular cartilage and subchondral bone microstructure in castrated PFJOA rat was recorded to a certain extent. Similarly, Guo et al.^87^ used selective COX2 inhibitors like imrecoxib and celecoxib to analyze changes in bone metabolism markers related to sacroiliac joint inflammation in patients with axial spondyloarthritis (axSpA) after treatment with COX2 inhibitors. As a result, increased levels of sclerostin, *Dkk1*, *Runx2*, and *OPG*, and decreased levels of β-catenin and BMP-2 in patients with axial spondyloarthritis were recorded. This alteration in marker proteins indicates a regulatory effect of BMPs and COX2 inhibitors on bone metabolism, syndesmophytes formation, and improvement of cartilage repair.

TGF-β and BMPs share a common mechanism of action through the activation of the Smad signaling pathway, which regulates gene expression related to osteoblast differentiation and bone formation.^88^ This overlap occurs because both TGF-β and BMPs belong to the TGF-β superfamily and thus utilize similar receptor systems and intracellular signaling cascades to promote anabolic processes in bone tissue to treat conditions like OA. However, their excessive release boosts OA progression and leads to synovial fibrosis as well as subchondral bone sclerosis. Zhu et al.^89^ achieved disease-modifying effects in early OA using pirfenidone (PFD), a TGF-β inhibitor. PFD achieved anti-inflammatory and anti-fibrotic effects across multiple joint tissues by reducing the expression of pro-fibrotic factors such as collagen I, fibronectin, and α-smooth muscle actin (α-SMA), while also decreasing IL-1β and IL-6 via downregulating Smad2/3 phosphorylation, which is crucial for TGF-β signaling activation. Another study by Zhu et al.^90^ reported that PFD blocked TGF-β2, which regulates fibroblast proliferation and ECM component overexpression in joint tissues by reducing the expression of fibrotic markers α-SMA and collagen types I and IV. By regulating genes associated with ECM synthesis, including *Col-1* and *Col-4*, PFD modulated the Wnt/β-catenin signaling pathway by inhibiting the phosphorylation of GSK-3β and downregulating heat shock protein 47 (HSP47), which is crucial for collagen processing. Similarly, Wei et al.^91^ showed that PFD delayed OA progression by inhibiting the proliferation and migration of FLSs stimulated by TGF-β1, while decreasing the expression *Col1a1*, *TIMP-1*, and *ACTA-2* (α-SMA), and IL-6 without affecting TNF-α levels, thereby alleviating synovial inflammation and fibrosis, as evidenced by improved OARSI scores in OA models. Besides, Lee et al.^92^ discovered SB431542, a TGF-β1 receptor inhibitor, reduced expression of *Col-1A*, connective tissue growth factor (CTGF), and α-SMA production while effectively mitigating TGF-β1-induced collagen gel contraction. When encapsulated in poly (D, L-lactide-co-glycolide) (PLGA) microspheres, SB431542 provided sustained local delivery, maintaining dose-dependent therapeutic concentrations that inhibit the fibrotic cascade in knee cartilage. Recently, Thielen et al.^93^ used low concentrations of the ALK4/5/7 kinase inhibitor SB-505124 to maintain TGF-β-induced Smad2/3 signaling, which is crucial for cartilage homeostasis, while blocking the harmful Smad1/5/9 pathway that contributes to chondrocyte hypertrophy and OA progression, as evidenced by decreased expression of *Runx2* and *Col10a1*. This selective inhibition of TGF-β receptors allowed for the induction of protective genes like *JunB* and *SERPINE1*, suggesting that the fine-tuning TGF-β receptor activation can separate the beneficial and detrimental effects of TGF-β in OA patients. In short, anabolic drugs targeting osteogenic growth factors hold promise for bone regeneration, but potential side effects and gaps in understanding long-term impacts necessitate further research to ensure their safety and efficacy.

#### Anti-resorptive agents

Anti-resorptive drugs inhibit bone resorption and slow down OA progression by maintaining bone density and integrity. These drugs, including bisphosphonates and RANKL inhibitors, target pathways such as the RANK/RANKL/OPG system and inhibit osteoclast activity, which is crucial for bone remodeling.^94^ By reducing bone turnover, they have shown promise in preclinical and early clinical studies for alleviating changes in subchondral bone and mitigating OA pain.

### Bisphosphonates

Bisphosphonates are a class of drugs that are structurally analogous to pyrophosphate, which allows them to bind to hydroxyapatite in bone to decrease osteoclast activity and prolong survival of osteoblasts. When the therapeutic effects of dose-dependent ibandronate were investigated in vitro and in vivo rat models of KOA, ibandronate inhibited TLRs/myeloid differentiation factor 88 (MyD88)/NF-κB signaling pathway. The study demonstrated reduced expression levels of TLR4, MyD88, and NF-κB and decreased inflammatory cytokines and apoptosis rates in both in vitro and in vivo models, associated with improved cartilage morphology, decreased pathological injury scores, and enhanced bone density parameters. Gielis et al.^95^ conducted the single-center, randomized, placebo-controlled, triple-blind ZODIAK trial included 86 patients aged 40 to 70 with symptomatic tibiofemoral OA to assess the efficacy of biannual infusions of zoledronic acid (ZA) in influencing cartilage thickness, joint space narrowing, and BML size over a 24-month period alongside clinical outcomes such as pain scores and quality of life measures. As a result, though clinical parameters showed some improvements within both the ZA and placebo groups via modulation of bone metabolism and inflammatory responses, potentially through pathways related to osteoclast activity and cytokine regulation, no significant differences were found between them. Additionally, prolonged use of bisphosphonates may lead to increased bone rigidity and microdamage accumulation, potentially exacerbating joint stress and accelerating OA progression. Nonetheless, the variability in patient responses and the presence of confounding factors in clinical studies have also contributed to inconsistent results regarding their efficacy in OA management.

### RANKL inhibitors

The RANKL pathway is a critical signaling cascade that regulates osteoclast differentiation and function essential for maintaining bone homeostasis. RANKL binds to its receptor RANK on osteoclast precursors to activate downstream signaling through TRAF6, which subsequently engages the NF-κB and MAPK pathways. This activation promotes the expression of *NFATc1* and *c-Fos*, essential for osteoclastogenesis and the expression of osteoclast-specific genes like *cathepsin K* and *TRAP*.^96^ Under OA conditions, excessive RANKL signaling contributes to increased osteoclast activity and results in enhanced bone resorption and joint degradation.^97^ Inhibiting the RANKL pathway with drugs like denosumab disrupts this interaction, prevents RANKL from binding to RANK, thereby blocking the recruitment of TRAF6 and subsequent activation of NF-κB and MAPK signaling and decreasing the expression of IL-1β and TNF-α. Shangguan et al.^98^ investigated the effects of denosumab on osteoclast activity and chondrocyte apoptosis in OA patients and found that denosumab effectively inhibited RANKL-induced osteoclastogenesis by blocking the RANK/RANKL signaling pathway via competitive bidding to RANKL. Consequently, reduced activation of downstream signaling pathways, particularly the NF-κB pathway, downregulated *cathepsin K* and *TRAP*, and mitigated ROS-induced chondrocyte apoptosis by regulating apoptotic gene expression were recorded, which ultimately contributed to reduce subchondral bone remodeling and cartilage degeneration in OA patients. Similarly, denosumab modified radiographic progression and decreased structural damage in erosive hand OA in a placebo-controlled, double-blind, randomized phase 2a clinical trial by acting as RANKL inhibitor by preventing the binding of RANKL to its receptor RANK on osteoclast precursors and reducing the expression of *NFATc1* and *TRAP*.^99^ Ongoing research on RANKL inhibitors aims to optimize the therapeutic index while minimizing the potential for adverse events to improve outcomes for patients with OA.

#### Vitamin D and calcium supplements

Though vitamin D and calcium supplements are not primary anti-resorptive agents, they are important components in OA management and support anti-resorption. When a case-control study examined the association between vitamin D status and oxidative stress markers, MMPs, and cartilage oligomeric matrix protein (COMP) in 124 patients with mild to moderate KOA and 65 healthy controls, Amirkhizi et al.^100^ found that individuals with vitamin D insufficiency had higher levels of markers of oxidative stress like malondialdehyde (MDA), total oxidant status (TOS), superoxide dismutase (SOD), and oxidative stress index (OSI), as well as lower levels of antioxidants like paraoxonase-1 (PON-1) and total antioxidant capacity (TAC). Serum vitamin D levels were inversely correlated with MDA, TOS, SOD, OSI, MMP1, and MMP13, and positively associated with TAC levels. Patients with sufficient vitamin D had lower MMP1 and MMP13 levels compared to those with insufficiency of vitamin D, suggesting that vitamin D promotes anti-resorption by upregulating antioxidant enzymes like PON-1 and TAC and downregulating MMPs like MMP1 and MMP13, to protect against cartilage degradation in OA. According to Busa et al.^101^, vitamin D in OA Wistar rats induced by anterior cruciate ligament transection (ACLT) combined with medial meniscectomy (MMx) reduced pain, inflammation, cartilage destruction, and MMP levels in a dose-dependent manner by decreasing C-telopeptide of type II collagen (CTX-II) levels, downregulating TNF-α, IL-1β, and IL-6, while upregulating anti-inflammatory IL-10. Furthermore, vitamin D treatment reduced serum levels of MMP3, MMP9, and MMP13, which contribute to articular cartilage destruction. In vitro studies showed that vitamin D (50-500 IU) significantly reduced mRNA levels of MMPs, NF-κB, TNF-α, and IL-6, while increasing IL-10 in IL-1β stimulated rat chondrocytes, indicating chondroprotective effects of vitamin D. Another RCT involving 240 KOA subjects with vitamin D deficiency was conducted and found that vitamin D supplementation significantly reduced knee pain and improved physical function, as evidenced by a marked decrease in VAS and WOMAC scores in the treatment group. The biochemical analysis revealed significant increases in serum 25(OH)D levels, calcium, and phosphate in the vitamin D group.^102^ Moreover, Sarig-Rapaport et al.^103^ conducted a pilot exploratory clinical study to evaluate the efficacy and safety of amorphous calcium carbonate (ACC) in treating canine OA. As a result, ACC counteracted the acidic conditions by increasing bicarbonate levels in the joint environment that promotes osteoclastic bone resorption and inflammation, thereby stabilizing subchondral bone remodeling. This modulation of pH is believed to influence the activity of acid-sensing ion channels (ASICs) and other nociceptive pathways that contribute to pain sensation and inflammatory responses. However, the detailed pathways of these agents are yet to be investigated in further trials to effectively treat OA conditions.

### Cartilage repair and regenerative therapies

Cartilage repair and regenerative therapies restore damaged cartilage and improve joint function in OA through various innovative approaches. They generally enhance cartilage regeneration by promoting chondrocyte proliferation, modulating inflammation, and improving the ECM environment. Key factors and pathways involved include growth factors such as TGF-β and IGF-1, signaling pathways like Wnt/β-catenin, and target genes associated with cartilage homeostasis, such as *Col2a1* and *Acan*.

#### Glucagon-like peptide (GLP-1, GLP-1R agonist)

Glucagon-like peptide-1 (GLP-1) and its receptor (GLP-1R) have demonstrated potential therapeutic roles in OA by modulating key cellular processes involved in joint degeneration.^104^ GLP-1R is expressed in chondrocytes, synoviocytes, and immune cells within joint, where its activation exerts anti-inflammatory effects by inhibiting the NF-κB pathway, thereby reducing pro-inflammatory cytokines and MMPs responsible for cartilage degradation. Additionally, GLP-1 signaling enhances rat chondrocyte viability by reducing apoptosis, promoting autophagy (via upregulation of *LC3-II* and *Beclin-1*), and attenuating oxidative stress through activation of the Nrf2/HO-1 antioxidant pathway. It also improves mitochondrial function by stimulating PGC-1α-mediated metabolic reprogramming. In the synovium, GLP-1 inhibits the activation of NLRP3 inflammasome and mitigates macrophage infiltration, limiting synovial inflammation and fibrosis. Moreover, GLP-1 agonist was reported to rescue type II collagen and aggrecan in human primary chondrocytes.^105^ Notably, GLP-1 agonists have been evidenced to significantly reduce knee pain in both clinical trials and animal studies.^106,107^ Collectively, these mechanisms highlight the potential of GLP-1/GLP-1R signaling as a multifaceted target for disease modification in OA, particularly in metabolically compromised individuals.^108,109^ Both GLP-1/GLP-1R signaling and 5-aminosalicylic acid (5-ASA) demonstrated promising anti-inflammatory and chondroprotective effects in OA models, highlighting their potential as disease-modifying agents. While GLP-1 acts through modulation of NF-κB signaling, reduction of oxidative stress, and enhancement of chondrocyte survival and autophagy, 5-ASA similarly downregulates inflammatory mediators (IL-6, COX-2, IL-8) and promotes anabolic responses, including increased proteoglycan synthesis and ECM preservation. These parallels suggest that targeting inflammation through diverse yet complementary pathways may offer synergistic opportunities for OA treatment.^41,107^

#### Hyaluronic acid

In a self-assembled hyaluronic acid (HA) and alpha-ketoglutarate (α-KG) NPs system, the benefits of the chondroprotective abilities of this system showed significant efficacy in relieving pain, enhancing mobility, and reducing cartilage damage in both early and advanced stages of OA.^110^ In a treated group of mice, NPs activated PERK-ATF4 signaling pathway, which alleviates endoplasmic reticulum stress (ERS) in chondrocytes and promoted cartilage matrix synthesis while inhibiting degradation. Specifically, αKG was shown to upregulate the expression of *Col2a1* and *Acan* while downregulating MMPs associated with cartilage degradation. Additionally, the high uptake efficiency of HA-αKG NPs by chondrocytes was linked to CD44 receptor interaction. When a single intra-articular injection of HA and HA/tranexamic acid (TXA) was administered to a murine model of KOA induced by MIA, Brochard et al.^111^ investigated that the therapeutic effects of HA/TXA are primarily involved in the role of TXA as a plasminogen activator inhibitor. Specifically, this complex mitigates fibrinolysis and subsequently reduces MMP activity. HA/TXA significantly improved pain thresholds compared to saline and HA alone, while both treatments exhibited chondroprotective effects by lowering OARSI scores. Similarly, Rasmussen et al.^112^ evaluated the long-term effects of intra-articular injections of gold microparticles using HA as a carrier for the treatment of KOA in 136 patients and found that 69% of patients reported a positive effect with improvements in pain, stiffness, and function as measured by the WOMAC index in a two-year follow-up study. This is due to the ability of HA to enhance the local delivery and retention of gold microparticles within the joint space. Gold microparticles larger than 20 μm were too large for macrophages to phagocytose, allowing them to slowly dissolve through a process called dissolucytosis, continuously exposing ECM and cells to therapeutic gold ions. However, the study identified that severe OA, obesity, and neuropathic pain (as indicated by a Pain Detect Questionnaire score ≥ 13) can reduce the efficacy of this treatment approach. However, HA-based treatments cannot completely reverse the multifaceted damages of advanced OA conditions, which require synergistic effects of therapies in the long run.

#### Platelet rich plasma

Platelet-rich plasma (PRP) is an innovative treatment method derived from autologous blood, characterized by a high concentration of platelets that promotes healing and tissue regeneration. Its effectiveness against OA, particularly in the knee, is attributed to its ability to modulate critical biological pathways such as Wnt and IL-1 signaling, which are implicated in cartilage degradation and inflammation.^113^ In an RCT, Patel et al.^114^ evaluated the efficacy of two different dosages of PRP injections, 4 mL and 8 mL, in patients with early KOA. The study involved patients with Kellgren-Lawrence (K-L) grades 1 and 2, who were divided into two groups receiving either dosage. The high concentration of platelets in PRP released TGF-β, VEGF, and platelet-derived growth factor (PDGF), which promoted tissue repair and regeneration by stimulating chondrocyte proliferation and matrix synthesis. As a result, 8 mL superdose of PRP significantly improved patient-reported outcomes compared to the 4 mL dose, likely due to the higher absolute platelet count (5.65 billion versus 2.82 billion), which correlates with enhanced regenerative capacity and sustained symptomatic relief over time.

#### Chondroprotective agents

Chondroprotective agents manage OA conditions by inhibiting MMPs, stimulating the synthesis of ECM components, promoting chondrocyte proliferation, and enhancing the production of collagen and proteoglycans. Key molecular pathways include the inhibition of TNF-α and IL-1β, along with the modulation of NF-κB, along with genes associated with cartilage homeostasis, such as those encoding for type II collagen and aggrecan, contributing to their protective effects against OA progression.^115^ Glucosamines, glycosaminoglycans, peptide complexes, and some natural compounds have been shown as effective chondroprotective drugs for treating OA symptoms.

##### Glucosamines

In a clinical trial, Ni et al.^116^ combined San-Bi-Tang with glucosamine sulfate (GS) capsules to treat cold-dampness-type KOA among 110 patients, who were randomly divided into a control group receiving only GS and an experimental group receiving both GS and San-Bi-Tang for five weeks. The findings indicated that the combination therapy significantly improved pain and joint function, as evidenced by reductions in VAS and WOMAC scores, alongside enhancements in knee joint performance metrics. GS promoted cartilage matrix synthesis, inhibited MMPs, modulated inflammatory pathways, and enhanced the expression of genes associated with cartilage health, including those encoding for type II collagen and aggrecan. To maintain steady supply of GS, Liu et al.^117^ developed polyethylene glycol-stabilized bilayer-decorated cationic liposome (CLis) as a novel drug delivery system to encapsulate GS for OA treatment in a rat model. The GS-CLis were synthesized using a reverse evaporation method, achieving an impressive encapsulation rate of 96.2% and a drug-loading capacity of 9.6%, which effectively improved the retention time and therapeutic efficacy of GS by facilitating its targeted delivery to chondrocytes and enhancing its anti-inflammatory and cartilage-protective effects.

##### Glycosaminoglycans

Xu et al.^118^ employed chondroitin sulfate-soluble undenatured type II collagen complex (CS-SC II) compared to soluble type II collagen (SC II) in a rat OA model to investigate its gastrointestinal digestive characteristics and therapeutic effects. CS-SC II exhibited superior gastric digestive stability at both pH 2.0 and pH 3.0, maintaining over 60% of its α1 chain and triple helix structure, while SC II was stable only at pH 3.0. In vivo, CS-SC II reduced joint swelling and improved weight-bearing ratios comparable to higher doses of SC II. CS-SC II enhanced the proportion of regulatory T cells (Tregs) in the spleen and downregulated mRNA expression of *IL-6*, *Ccl17*, *Mmp3*, and *Mmp13*. This modulation occurred through the inhibition of NF-κB and AKT signaling pathways. However, Chang et al.^119^ targeted NLRP3 inflammasome in both in vitro and in vivo OA models using chondroitin sulfate oligosaccharides (oligo-CS) by stimulating human SW1353 chondrocytes and THP-1 macrophages with octacalcium phosphate (OCP) crystals, which activated the NLRP3 inflammasome, leading to increased production of IL-1β and IL-6 as well as MMP13 and ADAMTS5. Oligo-CS treatment effectively inhibited the activation of the NLRP3 inflammasome and reduced the secretion of IL-1β, prevented downstream NF-κB pathway activation, and decreased the expression of catabolic genes. In vivo, oligo-CS administration maintained cartilage integrity and improved joint health in OA mice by modulating gut microbiota composition, which is linked to inflammatory responses.

##### Glycosaminoglycan-peptide complex (GP-C)

To enhance therapeutic delivery in OA treatments, Sodhi and Panitch^120^ developed a glycosaminoglycan (GAG)-based NPs system for the encapsulation of cationic peptides, specifically focusing on the KAFAK sequence. NPs formed through electrostatic interactions between the KAFAK peptide and sulfated hyaluronic acid (SHA) effectively protected the peptide from proteolytic degradation while allowing for controlled release over a pH range of 4.5 to neutral. In vitro experiments using inflamed keratinocytes revealed that the KAFAK-MK2i peptide complex significantly suppressed IL-6, IL-1β, and TGF-β. This suppression was attributed to the nanoparticles’ ability to inhibit the activation of the p38 MAPK pathway. Additionally, MALDI-TOF analysis confirmed that the encapsulated peptides retained structural integrity in the presence of enzymatic degradation, thus enhancing their biological efficacy. In an observational, non-interventional analysis of 9190 KOA patients, Karateev et al.^121^ evaluated the efficacy and safety of a glycosaminoglycan-peptide complex (GPC) combined with diacerein compared to GPC monotherapy. The combination therapy significantly reduced pain intensity at rest and during movement as well as improved overall health assessments, compared to GPC alone. GPC likely exerted its effects by modulating inflammation and cartilage degradation pathways. The combination therapy was also associated with a greater reduction in joint space narrowing (JSN), suggesting a potential disease-modifying effect.

#### MMPs inhibitors

The upregulation of MMPs, particularly MMP13, causes excessive cartilage breakdown and joint degeneration, while their downregulation by potentially therapeutic inhibitors relieves OA conditions and reverses these degenerative processes. Hu et al.^122^ utilized a GelMA-alginate hydrogel-based in vitro model to simulate OA conditions induced by IL-1β and TNF-α. The presence of these cytokines led to increased MMP13 activity, while treatment with MMP inhibitors resulted in a significant reduction in MMP13 levels and subsequent decreases in C2C fragment concentrations, which are markers of collagen degradation. This indicated that the inhibitors effectively blocked the enzymatic activity of MMP13 and preserved the ECM integrity. When an enzyme-responsive hydrogel was used to encapsulate BI-4394 against cartilage degeneration in OA model induced by ACLT in rats, Roy et al.^123^ proposed that BI-4394 effectively blocked enzymatic activity of MMP13 to reduce levels of collagen degradation markers, specifically C2C fragments. Histological evaluations revealed that treatment with the hydrogel significantly preserved cartilage structure and integrity compared to controls, evidenced by enhanced expression of collagen II and aggrecan while minimizing MMP13 levels. Similarly, Moqadami et al.^124^ investigated the protective effects of minocycline, an MMP inhibitor, against IL-1β-induced chondrocyte apoptosis and MMPs upregulation in human C28/I2 chondrocytes. The IL-1β exposure led to increased MMP13 expression, while minocycline treatment effectively inhibited the expression of pro-apoptotic factors such as *Bax* and *caspase-3*, and increasing the anti-apoptotic factor *Bcl-2*, and reduced the number of apoptotic cells. Additionally, minocycline suppressed MMP13 expression, thereby mitigating the degradation of type II collagen in the extracellular matrix. These effects were linked to the inhibition of the NF-κB signaling pathway, activated by IL-1β.

However, despite this strong molecular evidence, the translation of MMPs inhibitors into effective clinical therapies for OA has been not successful. The clinical failure of MMPs inhibitors is attributed to several key factors. For instance, the pan-MMPs inhibitors demonstrated severe off-target side effects due to a lack of specificity.^125^ A total of 12 patients were found to have developed cumulative toxicity following the administration of broad-spectrum MMPs inhibitors.^126^ This underscored the critical need for highly selective agents, such as MMP13 inhibitors. However, even specific MMP13 inhibition faces the challenge of side effects, functional redundancy, and limited therapeutic efficacy. The clinical failure of MMP13 inhibitors, despite strong preclinical potential, is attributable to a confluence of functional redundancy, various proteoforms, and the lack of precision therapy strategies. A fundamental challenge is the functional redundancy within the MMP family.^127^ Other collagenases with similar functions, such as MMP1 and MMP8, can compensate for inhibited MMP13, perpetuating cartilage degradation. There is limited insight into the network of interactions between MMPs and inhibitors in biology.^128^ Secondly, various post-translational modifications (PTMs) are also a key factor. MMP13 exists as complex protein mixtures due to PTMs like glycosylation and methylation. These modifications alter enzyme activity and conformation, meaning an inhibitor designed for one form may be ineffective against others. Furthermore, a significant disconnect exists between preclinical models and human OA. The pathophysiology of human OA is a chronic process that fundamentally differs from the post-traumatic pathology modeled in young animals. Moreover, side effects remain a persistent challenge for selective inhibitors. It has been reported that PF152 (MMP13 inhibitor) showed severe nephrotoxicity.^128,129^ Lastly, MMP13 is not the sole factor responsible for cartilage destruction, as OA is a highly heterogeneous disease.^130^ In patients exhibiting a primary characteristic of proteoglycan loss, the inhibition of MMP13 alone has been demonstrated to be ineffective in preventing cartilage degeneration.^131^ Consequently, the absence of patient stratification emerges as a pivotal factor in the failure of clinical trials. In the absence of precision medicine, the population-wide mean efficacy is calculated, which can lead to the erroneous conclusion of ineffectiveness. Advancing MMP therapy requires navigating its biological complexity and improving drug specificity. The development of advancing proteomic techniques and tests for net MMP activities enables precision medicine in OA. In conclusion, MMP inhibitors remain a promising medicine for OA.

#### AMPK activators

Systemic metabolic dysregulation has emerged as a pivotal, load-independent driver in the pathogenesis of OA. These disorders, including obesity and type 2 diabetes, create a pro-inflammatory and metabolically disordered microenvironment within the joint.^132^ 5’-AMP activated protein kinase (AMPK) is a key regulator of cellular metabolism and energy balance in many aging-related diseases, such as type 2 diabetes, cancer, hyperlipidemia and OA.^133^ OA-associated reductions in AMPK activity have been observed in mice and human chondrocytes and cartilage.^36^ This reduced activity contributes to autophagy and mitochondrial dysfunction in OA chondrocytes. Preclinical research has identified A-769662 as a pharmacological AMPK activator with the potential to rescue deficient mitochondrial biogenesis in OA chondrocytes.^134^ Moreover, natural products such as Ginsenoside Rh1 and curcumin can combat OA by promoting AMPK-mediated mitophagy in chondrocytes.^135,136^ AMPK activators not only alleviate OA through chondrocytes but also exert effects by inhibiting synovitis, pain, and subchondral bone sclerosis in non-human primates. For example, chitosan oligosaccharide inhibited inflammation in both primary rabbit and human synoviocytes by activating AMPK.^36^ AICAR and metformin, two well-known AMPK agonists, were reported to inhibit osteogenic differentiation and relieve pain level in mouse and rhesus macaques models.^137,138^ Notably, a handful of clinical trials related to OA treatment have been conducted on metformin in the past two years. Pan et al. found that metformin demonstrated significant improvement in knee pain among overweight or obese OA patients in a randomised controlled trial.^139^ Although the effect size was moderate (SMD = 0.43) and larger trials are needed for confirmation, these results provide direct clinical evidence in support of the concept of treating OA by targeting AMPK. This collective evidence indicates that AMPK is pivotal in the alleviation of OA pain and the improvement of cartilage damage in both animal models and clinical trials. This stands in contrast to the outcomes of targeting other metabolic pathways, such as mTOR. A study revealed that in guinea pig models, oral rapamycin-induced mTOR inhibition (even combined with metformin to activate AMPK) exacerbated OA by inducing hyperglycaemia.^140^ This finding suggests that mTOR inhibitors may carry unforeseen metabolic risks. Consequently, AMPK activators represent a promising and well-validated class of therapeutic candidates for OA.

### Senolytic and senomorphic agents

**Fig. 4:**
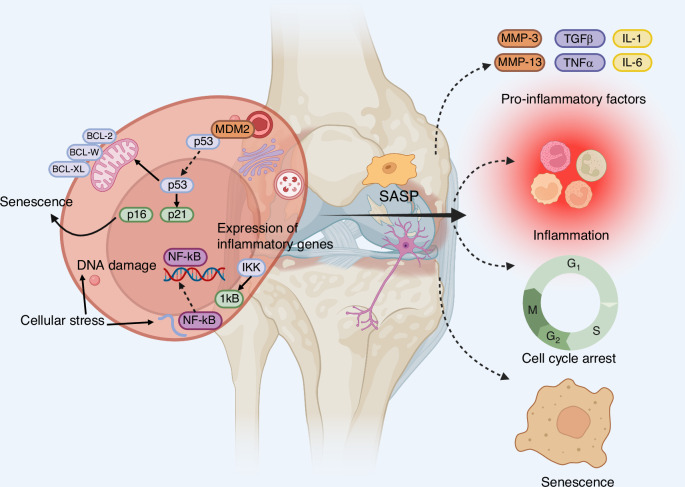
Cellular senescence promotes OA progression by activating p53-p21-p16 signaling, NF-κB–mediated inflammatory gene expression, and SASP-driven tissue degradation. Abbreviations: BCL-2 B-cell lymphoma 2, BCL-W B-cell lymphoma-W, BCL-XL B-cell lymphoma-extra large, p53 tumor protein p53, p21 cyclin-dependent kinase inhibitor 1, p16 cyclin-dependent kinase inhibitor 2 A, MDM2 mouse double minute 2, IKK IκB kinase, SASP senescence-associated secretory phenotype, TGF-β transforming growth factor-beta, G₁/S/G₂/M cell-cycle phases

Senolytic agents focus on the selective elimination of senescent cells (SCs) and the enhancement of cellular function to combat age-related OA progression.^141^ These drugs target pathways associated with cellular senescence, such as the p53 and p16INK4a pathways, which regulate cell cycle arrest and apoptosis. To remove senescent cells, these treatments induce apoptosis in SCs by inhibiting anti-apoptotic pathways mediated by Bcl-2 family proteins (e.g., Bcl-2 and Bcl-xL) and reduce the senescence-associated secretory phenotype (SASP) within senescent chondrocytes and synovial cells within the joint microenvironment, as shown in Fig. 4.^142^ SASP refers to the pro-inflammatory mix of cytokines, proteases, chemokines, and growth factors released by senescent cells that accelerates cartilage degradation, chronic inflammation, and OA progression. The key factors are growth factors like FGF and IGF-1, as well as signaling pathways that promote cellular repair and regeneration. Escriche-Navarro et al.^143^ developed mesoporous silica nanoparticles (MSNs) loaded with the senolytic navitoclax and coated with a specific peptide substrate of MMP3 (NPs(Nav)@MMP3). These nanoparticles preferentially released their cargo in ionizing radiation (IR)-induced senescence (SC) compared to proliferating cells, depending on MMP3 levels. Treatment with NPs(Nav)@MMP3 selectively decreased the viability of SCs and provided a protective effect on non-proliferating cells with a 5-fold increase in the senolytic index compared to free navitoclax. The NPs targeted the altered senescent microenvironment characterized by elevated MMP3 levels to trigger the selective death of SCs while limiting off-target effects on non-senescent cells. Wang et al.^144^ utilized DMM model in rats and IL-1β-treated chondrocytes to investigate navitoclax’s mechanisms. Navitoclax, as a senolytic agent, induced apoptosis in senescent chondrocytes through inhibition of anti-apoptotic Bcl-2 family proteins, decreased expression of senescence markers such as *p16INK4a* and *p21*, along with a reduction in SASP, and facilitated restoration of ECM homeostasis by promoting the synthesis of ECM components while inhibiting degrading enzymes like MMP13 and ADAMTS5.

Similarly, senomorphic agents modulate the behavior and characteristics of senescent cells to effectively restore their function to resemble that of younger, healthier cells. Under OA conditions, these agents target PI3K/AKT/NF-κB and YAP-1 pathways, promote chondroanabolism, stimulate the expression of *Sox9*, *Col2a1,* and *Acan* while simultaneously inhibiting catabolic processes mediated by MMPs and ADAMTSs to alleviate OA progression.^145^ Notably, both senomorphic and senolytic agents share a common goal of addressing cellular senescence; however, while senolytics focus on the selective elimination of senescent cells, senomorphics aim to rejuvenate their functionality without necessarily inducing cell death. Dhanabalan et al.^146^ developed rapamycin-loaded poly (lactic-co-glycolic acid) microparticles (RMPs) for treating mice with articular OA and found that RMPs effectively induced autophagy, as evidenced by increased levels of LC3B and reduced the expression of the senescence marker *p16INK4a*. The mechanism underlying these effects involves the inhibition of mTOR pathway and the production of sulfated glycosaminoglycans (sGAG) while decreasing inflammatory markers associated with OA pathology. The sustained release of rapamycin from MPs ensured prolonged therapeutic concentrations within the joint space for effective mitigation of cartilage damage and inflammation when administered intra-articularly. However, according to Luo et al.^147^, rapamycin, an mTOR inhibitor, effectively mitigated IL-1β-induced chondrocyte apoptosis and inflammation via upregulation of LC3B, downregulation of apoptotic markers including cleaved-caspase-3, and activation of PI3K/Akt signaling pathway. Additionally, rapamycin reduced the expression MMP3 and MMP13 while enhancing the production of collagen II and aggrecan. Similarly, metformin is a great option as a senomorphic agent that effectively mitigates OA conditions.

## Conclusion and future perspectives

OA is increasingly recognized as a complex, multifactorial disease that extends beyond mere cartilage degradation to involve the entire joint structure, including subchondral bone, synovium, and surrounding tissues. Despite significant advancements in our understanding of OA pathophysiology, including the identification of key molecular pathways and genetic factors, considerable gaps remain in elucidating the precise mechanisms underlying disease initiation and progression. The aging population globally, particularly in regions such as China, where projections indicate that nearly 22% of the population will be over 65 by 2033, underscores the urgent need for effective therapeutic strategies to manage OA.^1^ Currently, there is no approved DMOAD that can halt or reverse the progression of OA. Recent research has discovered various pathways involved in OA, including inflammation, cartilage metabolism, and subchondral bone remodeling. These insights pave the way for the development of novel therapeutic agents that could potentially modify disease progression rather than merely alleviating symptoms. However, the heterogeneity inherent in OA complicates treatment strategies; a one-size-fits-all approach is unlikely to be effective. Instead, there is a growing consensus on the need for personalized medicine approaches that consider individual patient characteristics, including genetic profiles and specific OA phenotypes. Improved patient stratification based on these factors could enhance the efficacy of emerging therapies and facilitate more targeted clinical trials.

As we move forward, several open questions remain. How can we better identify early-stage OA patients who are most likely to benefit from DMOADs? What biomarkers can reliably predict disease progression and treatment response? Furthermore, as new pharmacological agents enter clinical trials, it is essential to establish robust endpoints that reflect meaningful improvements in joint function and quality of life for patients. The integration of advanced imaging techniques and machine learning algorithms may provide valuable tools for identifying distinct OA phenotypes and tailoring therapeutic interventions accordingly. Ultimately, the goal is to develop effective treatments that not only address symptoms but also modify disease progression, thereby reducing the substantial social and economic burden associated with OA. Future perspectives in OA management are increasingly influenced by advances in technology and precision medicine. Computer and AI-aided drug development is revolutionizing the identification of new therapeutic targets by analyzing large datasets and predicting drug efficacy, reducing the time and cost of developing effective treatments. High-throughput drug screening complements this by enabling rapid testing of thousands of compounds, accelerating the discovery of promising DMOADs. Innovations in treatment approaches, such as tissue engineering, gene therapy, and advanced biomaterials, offer potential for regenerating cartilage and restoring joint function. Personalized medicine is emerging as a transformative strategy, tailoring therapies based on genetic, molecular, and lifestyle factors unique to each patient, enabling targeted interventions that minimize side effects and optimize outcomes. For example, integrating patient-specific phenotypes and biomarker profiles into therapeutic decision-making represents an essential future direction for precision OA management. However, advancing this approach will require treatment frameworks that match dominant disease mechanisms with mechanism-specific interventions. Targeted anti-cytokine strategies should be further developed for inflammatory phenotypes while metabolic phenotypes warrant structured weight-management programs and interventions that correct metabolic dysregulation. Mechanical phenotypes highlight the need for therapies that optimize joint load distribution and address alignment abnormalities. In addition, expanding senolytic and senescence-modulating strategies will be critical for patients exhibiting features of cellular senescence. Establishing phenotype-guided treatment algorithms has the potential to enhance therapeutic precision, improve clinical outcomes and minimize exposure to ineffective or non-essential interventions, ultimately advancing OA care toward a fully personalized model. These advancements, combined with a deeper understanding of OA pathophysiology, promise a future where OA management is both more effective and patient-centered.

In future, several challenges must be addressed to translate mechanistic insights into clinically meaningful therapies. For instance, a major limitation is the current lack of validated biomarkers that can reliably predict therapeutic response, stratify patients by dominant disease pathways, and guide treatment selection. Additionally, leveraging AI-assisted drug discovery and AI-driven patient stratification will require standardized data integration frameworks capable of handling multimodal clinical, imaging, and molecular datasets. Future clinical trials must also evolve toward adaptive designs that account for OA heterogeneity and enable real-time modification of interventions based on biomarker-defined subgroups. These forward-looking strategies are essential to accelerate the development of precision DMOADs and ensure that emerging therapies are beneficial for patients in long term.

In conclusion, while significant strides have been made in understanding OA pathophysiology, much work remains to be done to translate this knowledge into effective clinical therapies. The recognition of OA as a heterogeneous disease involving multiple joint tissues necessitates a paradigm shift in how we approach treatment. Current therapies primarily focus on symptom management; however, there is an urgent need for DMOADs that can effectively alter disease progression and improve long-term outcomes for OA patients. The exploration of novel therapeutic agents targeting inflammation, cell metabolism, and cellular senescence holds promise for reshaping the landscape of OA management. Moreover, as research continues to uncover the complex interactions between mechanical factors and biological processes in OA pathogenesis, future therapeutic approaches must consider both aspects holistically. As we look ahead, interdisciplinary collaborations among researchers, clinicians, and industry stakeholders will be vital in driving innovation and ensuring that emerging therapies translate into meaningful benefits for those affected by OA.
